# Aerobic exercise, but not isometric handgrip exercise, improves endothelial function and arterial stiffness in patients with myocardial infarction undergoing coronary intervention: a randomized pilot study

**DOI:** 10.1186/s12872-021-01849-2

**Published:** 2021-02-17

**Authors:** Daniel P. Kollet, Ana Beatriz Marenco, Nathan L. Bellé, Eduardo Barbosa, Liliana Boll, Bruna Eibel, Gustavo Waclawovsky, Alexandre Machado Lehnen

**Affiliations:** Institute of Cardiology of Rio Grande Do Sul/University Foundation of Cardiology, Unidade de Pesquisa, 3ºAndar, Av. Princesa Isabel, 395 Santana, Porto Alegre, RS 90620-001 Brazil

**Keywords:** Myocardial infarction, Endothelial function, Arterial stiffness, Aerobic exercise, Isometric handgrip exercise

## Abstract

**Background:**

Aerobic exercise improves endothelial function and arterial stiffness after myocardial infarction (MI), but the effects of isometric exercise on cardiovascular parameters are still uncertain. We aimed to assess the effects of one session of aerobic or isometric exercise on flow-mediated dilation (FMD) and pulse wave velocity (PWV) in post-MI volunteers undergoing percutaneous coronary intervention (PCI).

**Methods:**

Twenty post-MI patients undergoing PCI were randomized to aerobic (AE, n = 10) or isometric (IE, n = 10) exercise groups. We evaluated cardiac structure and function (echocardiographic); carotid plaque presence (ultrasound). FMD and PWV were measured 10 min before and 10 min after the intervention: a single session of moderate-intensity AE (30 min; ratings 12–14 on Borg’s scale or 50–60% HRreserve) or handgrip IE (four two-minute bilateral contractions; 30% maximal voluntary contraction; 1-min rest). Generalized estimating equations (Bonferroni post-hoc) was used to assess differences (*p* ≤ 0.050).

**Results:**

FMD improved only in the AE group (Δ = 4.9%; *p* = 0.034), with no difference between groups after exercise. Even after adjustment (for baseline brachial artery diameter) the effectiveness of AE remained (*p* = 0.025) with no change in the IE group. PWV was slightly reduced from baseline in the AE group (Δ = 0.61 m/s; *p* = 0.044), and no difference when compared to the IE group. Peripheral vascular resistance decreased in AE versus IE (*p* = 0.050) and from baseline (*p* = 0.014).

**Conclusions:**

Vascular measurements (FMD and PWV) improved after a single session of AE. There are apparently no benefits following a session of IE.

**Trial registration:**

http://www.clinicaltrials.gov and ID number NCT04000893.

## Background

Cardiovascular diseases including myocardial infarction (MI) are a leading cause of death worldwide. They accounted for 31% of all deaths globally in 2016 [1], and 85% of these deaths are due to acute MI and stroke [1].

Endothelial dysfunction is a characteristic feature that precedes the development of atherosclerosis [2] and asymptomatic structural vascular changes [3] as well as clinical manifestations of acute MI. On the other hand, changes in the middle layer of arteries are associated with arterial stiffness and major cardiovascular outcomes such ST-elevation MI [4]. Thus, the assessment of both endothelial function and arterial stiffness may help prevent MI and/or recurrent infarctions.

Flow-mediated dilation (FMD) is a method that assesses endothelial function by measuring changes in arterial diameter in response to hyperemia [5] and it is an indirect measure of the risk for cardiovascular events, including MI [6, 7]. Pulse wave velocity (PWV) is a method used to detect changes in the middle layer of arteries and PWV measures are associated with vascular stiffness [8].

Aerobic exercise (AE) has become an integral part of cardiac rehabilitation programs [9–12] while more evidence of vascular and hemodynamic benefits of other exercise modalities such as strength training is needed. According to the Physical Activity Guidelines for Americans, older adults should do multicomponent physical activity combining aerobic, muscle-strengthening and balance training [13]. Also, strength training is extremely important because it helps improving the ability to perform activities of daily living. As for its effects on the artery, low- to moderate-intensity strength training apparently improves endothelium-dependent vasodilation and decreases arterial stiffness [14–16] while high-intensity strength training seems to induce greater arterial stiffness [17]. Yet, these effects have been associated with long-term adaptations in healthy populations.

As for isometric exercise (IE) in particular, studies have shown that a single IE session was able to induce an hypotensive effect in individuals with hypertension [18, 19] but this benefit has not been evidenced in other studies [20]. Regarding endothelial function, a single exercise session decreased FMD [21], and although this impairment may be interpreted as harmful to individuals with hypertension, these effects appear transient since eight weeks of IE improved resting FMD in this population [22]. Regarding arterial stiffness, there is evidence showing increased PWV during isometric handgrip exercise [23] and after one exercise session [24]. One randomized clinical trial that assessed the effects of a single IE session in patients with coronary artery disease reported increased PWV [25].

Given that (1) strength training have health benefits [26], (2) isometric handgrip is a form of exercise easily applicable for muscle strengthening, but (3) cardiovascular benefits of isometric handgrip exercise are not entirely understood in post-acute MI individuals (who have several risk factors for cardiovascular diseases and reduced cardiac function) and (4) evidence on this exercise modality is scarce, in particular in individuals post-acute MI, we conducted a randomized pilot study to compare the effects of one session of isometric handgrip exercise and one session of AE on FMD and PWV in volunteers undergoing percutaneous coronary intervention (PCI) after acute MI. Our hypothesis was that one single IE session is enough to improve FMD and PWV, and that one single session of either IE or AE have similar effects in this population.

## Methods

We conducted a randomized, evaluator-blind, parallel‐group controlled pilot study following the Consolidated Standards of Reporting Trials (CONSORT) guidelines [27] and the principles of the Declaration of Helsinki. The research project was approved by the research ethics committee at Instituto de Cardiologia do Rio Grande do Sul/Fundação Universitária de Cardiologia (ICFUC) (protocol number 5326/17) and registered at ClinicalTrials (ID number NCT04000893; Registered 27 June 2019—Retrospectively registered, http://www.clinicaltrials.gov). All volunteer participants read and signed a free informed consent form.

### Study population and sample

The study sample was drawn from a list of patients who underwent primary PCI with second-generation stent placement at ICFUC hemodynamics service. Patients were invited to participate in the study through a phone call and asked to come to ICFUC Clinical Research Laboratory for a first visit (visit 1) when they were explained the study objectives and screened for eligibility. They were also told it was a requirement to attend four visits (2 to 7 days apart). Those who agreed to participate signed a free informed consent form. A medical questionnaire and the International Physical Activity Questionnaire (IPAq)—long version (http://www.ipaq.ki.se) were administered and information on alcohol consumption was collected. Heavy consumption was defined as > 15 (males) or > 10 (females) drinks/week (one drink contains 15 g of alcohol and is equivalent to 350 mL of beer, 150 mL of wine or 40 mL of spirits). Anthropometric measurements (height, body mass and waist circumference) were also taken.

The study sample comprised male and female patients aged 18 to 80 years diagnosed with acute MI with ST-elevation who underwent primary PCI within 48 h. We set a 48-h time frame based on Schomig et al*.* (2005) finding that patients with acute MI undergoing delayed PCI (within 12–48 h) showed good prognosis. [28] ICFUC is a reference cardiology center that provides care to patients that have to travel long distances for treatment. The other inclusion criteria were: being clinically and hemodynamically stable with left ventricular ejection fraction > 40% (calculated by Simpson method); and regular medication use including β-blockers, statins, acetylsalicylic acid (ASA), angiotensin-converting enzyme (ACE) inhibitors or angiotensin II receptor blockers (ARBs); and antiplatelet agents (clopidogrel). We excluded those with a history of other acute MI events; lesions in the coronary artery trunk evidenced in cardiac catheterization; pericarditis; complex supraventricular or ventricular arrhythmias; significant electrical conduction disturbances on ECG recordings; pulmonary emboli/thrombophlebitis; syncope or transient ischemic attack (TIA); intracardiac thrombi; blood pressure levels greater than 170/100 mmHg; any kind of thrombolytic therapy; and any orthopedic, physical or mental impairments that prevent physical exercise.

**Fig. 1:**
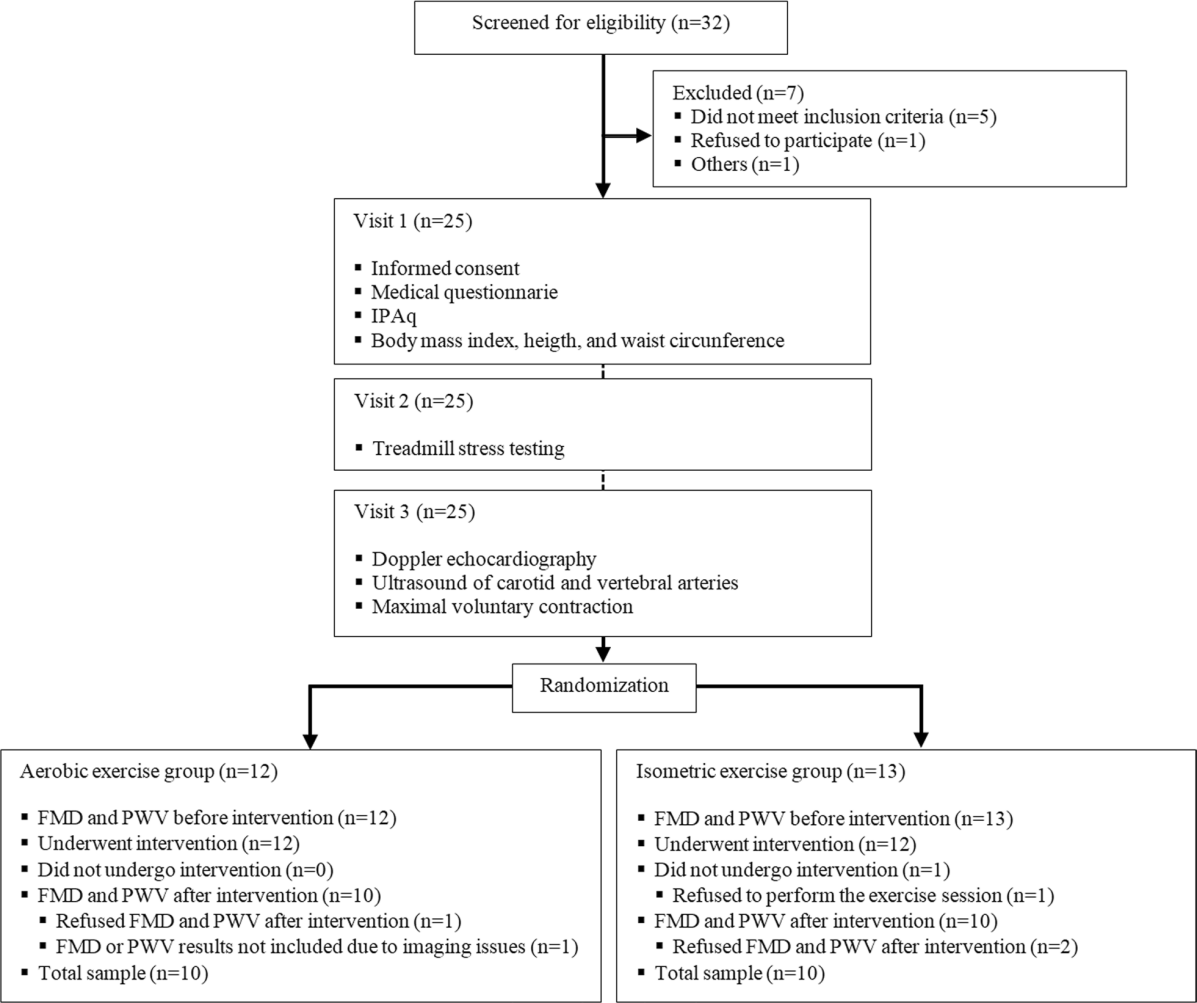
Study design. AE, aerobic exercise session; IE, isometric exercise session; PWV, pulse wave velocity; FMD, flow-mediated dilation of the brachial artery

The sample size was calculated based on Siasos et al*.* (2016). For a 5% significance level, 80% power and an expected (absolute) difference of 2.5% in FMD values following a typical aerobic exercise session, a sample of 24 participants (n = 12 per group) was estimated. Group allocation is detailed in Fig. 1.

The random allocation of participants was carried out by a blind collaborator using automatic code generation (http://www.randomization.com). Participants were assigned to two intervention groups: one session of aerobic exercise (AE) or one session of isometric handgrip exercise (IE). They were initially blinded to allocation groups. The study investigators that conducted patient evaluations were also blinded to group allocation.

### Exercise stress test

The volunteers underwent a submaximal exercise stress test (70–85% HRmax) to evaluate whether they were fit to participate in the study. The test was carried out on visit 2 within 20–25 days of hospital discharge in the morning hours (11 am to 12 pm). They were instructed to refrain from smoking, alcoholic and caffeinated beverage consumption and moderate to intense physical exertion within 24 h of exercise stress test and continue taking their medication. It was performed at ICFUC stress testing laboratory using a treadmill (Inbramed, Porto Alegre, Brazil) and ErgoPC 13 software according to Bruce protocol or a ramp protocol based on to the patient's profile and a previously established testing routine.

### Doppler echocardiography and ultrasound of carotid and vertebral arteries

On visit 3 (three days after the exercise stress test), the participants underwent imaging assessments at ICFUC Clinical Research Laboratory in the morning hours (8 am to 12 pm). They were asked to continue taking their medication. The same evaluator (D.K.) blind to intervention allocation performed all examinations using a Philips Medical Systems’ EnVisor ultrasound system (Bothell, WA/USA). Left ventricular (LV) volumes were measured using Simpson’s rule formula (biplane method). Left ventricular end-systolic volume (ESV) and end-diastolic volume (EDV) were adjusted for body surface area (ESVI and EDVI). We assessed the following transthoracic echocardiographic parameters: ventricle and atrium sizes; wall thickness and contractile function; valve structure and functions; large vessels (aorta, pulmonary arteries, vena cava, and pulmonary veins); segmental and global systolic and diastolic function; and pulmonary artery and right atrium pressure [30].

The same evaluator (N.L.B.) blind to intervention allocation performed all ultrasound examinations of carotid and vertebral arteries. The extracranial segments of right and left carotid arteries were examined for atherosclerotic plaques. Common, internal and external carotid arteries were examined using color Doppler flow imaging and flow velocities were measured using pulsed Doppler ultrasound. We assessed carotid artery stenosis based on the 2003 Consensus [31] by analyzing peak systolic velocity (PSV), internal carotid artery (ICA), common carotid artery (CCA) and end-diastolic velocity (EDVe) as follows: (1) < 50% carotid stenosis: ICA PSV < 125 cm/s; carotid plaque with < 50% cross-sectional luminal narrowing; (2) 50%–69% carotid stenosis: ICA PSV 125–230 cm/s; plaque with ≥ 50% cross-sectional luminal narrowing; (3) ≥ 70% carotid stenosis: ICA PSV > 230 cm/s; plate with > 50% cross-sectional luminal narrowing; (4) carotid sub-occlusion: PSV variables; plate with large carotid sub-occlusion; (5) carotid occlusion: no detectable patent lumen and flow. Other additional parameters were also assessed: (1) < 50% carotid stenosis: PSV to ICA/CCA ratio < 2 and EDVe < 40 cm/s; (2) 50%–69% carotid stenosis: PSV to ICA/CCA ratio 2 to 4 and EDVe 40–100 cm/s; (3) ≥ 70% carotid stenosis: PSV to ICA/CCA ratio > 4 and EDVe > 100 cm/s.

### Maximal voluntary contraction

Maximal voluntary contraction (MVC) was assessed on visit 3 (ICFUC Clinical Research Laboratory) in the morning hours (8:30 to 11:30 am). The participants were asked not to change their eating habits and sleep hours and continue taking their medication. An analog hand dynamometer was used to measure isometric grip strength (Jamar, Chicago, USA). The participants held a handgrip device while sitting upright with feet flat on the floor and at the evaluator’s command performed a single maximal contraction of the hand flexor muscles with each hand, one at a time, to determine MVC. Three attempts were made with each hand, with intervals of 3 min between each, and the highest MVC values were recorded for each hand.

### Brachial artery flow-mediated dilation

Endothelial function of the brachial artery was assessed non-invasively using a high-resolution ultrasound device (Esaote MyLab™ 70 XVision; Genoa, Italy) 10 min before and 10 min after each exercise session in the morning hours (8:30–9 am) at the study laboratory. The participants were instructed to refrain from smoking, alcoholic and caffeinated beverage consumption and moderate to intense physical exertion within 24 h of FMD assessment and continue taking their medication. Longitudinal images of brachial artery walls were captured with a high frequency transducer at 7–12 MHz according to Thijssen et al*.* (2011). Doppler ultrasound video signals were captured at each step by a USB video card (EasyCAPture; China) connected to a computer for simultaneous recording and subsequent offline processing using a detection software (Cardiovasculare Suit™, Pisa, Italy). Baseline video signals were recorded for 1 min. Then, a fast deflation cuff (Incoterm™ EC500; Porto Alegre, Brazil) was placed on the participant’s forearm 5 cm distal to the cubital fossa and inflated to a pressure of 200 mmHg for 5 min. After cuff deflation, brachial artery video signals were recorded for 3 min. Blood flow was calculated at 30 Hz from synchronized arterial diameter and velocity data. FMD was calculated as the relative percent change in peak diameter following cuff deflation relative to the preceding baseline diameter.

### Pulse wave velocity

Central and peripheral blood pressure and PWV were measured 10 min before and 10 min after each intervention and immediately after FMD measurements using a validated oscillometric blood pressure measurement device (Mobil-O-Graph 24 h PWA Monitor®, IEM GmbH, Stolberg, Germany) [33, 34] at ICFUC Clinical Research Laboratory. With the cuff placed around the participant's arm, three automated readings were made at intervals of 3 min. The instrument measured and calculated central systolic blood pressure (cSBP), central diastolic blood pressure (cDBP) and PWV. PWV estimates were derived from in-built ARCSolver algorithms.

### Study intervention: aerobic and isometric sessions

All exercise sessions were held on visit 4 (ICFUC Clinical Research Laboratory), 3–5 days after visit 3. The participants were instructed to refrain from smoking, alcoholic and caffeinated beverage consumption and moderate to intense physical exertion within 24 h of the intervention and continue taking their medication. The AE intervention was performed on an exercise bike (Movement BM4500pró, São Paulo, Brazil). After a 5-min warm-up the participants performed 30 min of moderate-intensity exercise measured by the Borg Rating of Perceived Exertion (RPE) scale (scores 12 to 14) [35]. According to The American College of Sports Medicine, moderate intensity is achieved at 50–60% of HRreserve [36] and a rating of 12–14 on the Borg scale [35]. In addition to RPE, we also assessed HR during AE session, defined as HRexercise = (HRmax − HRrest) × intensity + HRrest [36]; HRrest was the lowest HR lying down in a supine position for 20 min before FMD; HRmax was calculated using the following equation: 220—age. Given a mean age of 58.6 years in the AE group and a HRrest of 70 bpm, mean HRexercise ranged from 116 bpm (50% HRreserve) to 125 bpm (60% HRreserve). Since most participants in our sample were on beta-blockers, we primarily used Borg ratings and HRreserve as an adjuvant to quantify exercise intensity during AE session. They finished the session with a 3-min cooldown. All HR data were collected with the use of a heart monitor (POLAR™ RS800CX RUN, Helsinki, Finland).

The IE intervention was performed using an analog handgrip device (Jamar hand dynamometer, Chicago, USA). The participants held the handgrip while sitting upright with feet flat on the floor and four, 2-min alternating bilateral contractions of the hand flexor muscles at 30% MVC with 1-min rest between contractions, totaling 23 min of contraction as described in the literature [21, 38]. They were asked to keep their elbows flexed to 90° with the handgrip lightly resting on their leg during the entire exercise session. They were given feedback and encouraged to sustain at 30% MVC and avoid the Valsalva maneuver during contractions.

### Statistical analyses

The Shapiro–Wilk test was conducted to assess data distribution. Mean ± standard deviation and/or 95% confidence intervals (95% CI) were used to describe the sample data. Differences between AE and IE groups regarding demographic characteristics, cardiovascular risk factors, laboratory test results (Table 1), echocardiographic and carotid measurements (Table 2), medication use (Table 3) and MI clinical features (Table 4) were assessed with the use of Student's t-test for independent samples or the chi-square test when applicable. Differences in FMD measurements (baseline diameter, peak diameter and %FMD), central and peripheral blood pressure values, PWV and peripheral vascular resistance (PVR) were analyzed using generalized estimating equations (GEE) for two factors (groups and times and the interaction between them) with Bonferroni post-hoc test. The statistical significance was set at *p* ≤ 0.05 for all tests. Data were analyzed using SPSS Statistics version 24 (Chicago, IL, USA).

**Table 1:** Participants’ demographic characteristics, cardiovascular risk factors and laboratory results

	AE (n = 10)	IE (n = 10)	*p* value
Age (years)	58.6 ± 9.0	56.0 ± 9.8	0.543
Gender			0.531
Female (n)	1	2	
Male (n)	9	8	
Ethnicity			0.136
White (n)	8	10	
Black (n)	2	0	
Education			0.448
Elementary incomplete (n)	2	3	
Elementary complete (n)	1	0	
High school incomplete (n)	0	2	
High school complete (n)	5	3	
College complete (n)	2	2	
Hypertension prior to acute MI			0.329
Yes (n)	8	6	
No (n)	2	4	
Physical activity level			0.361
Low (n)	5	7	
Moderate or high (n)	5	3	
Family history of CAD			0.531
Yes (n)	9	8	
No (n)	1	2	
Diabetes mellitus			0.329
Yes (n)	4	2	
No (n)	6	8	
Excessive alcohol use*			0.051
Yes (n)	5	1	
No (n)	5	9	
Smoking			0.178
Yes (n)	7	4	
No (n)	3	6	
Anthropometric measurements and laboratory values (mean ± SD)			
Total body weight (kg)	79.1 ± 8.1	79.4 ± 17.5	0.961
BMI (kg/m^2^)	28.6 ± 1.9	28.3 ± 5.0	0.855
Waist circumference (cm)	95.3 ± 3.4	94.5 ± 9.3	0.802
Total cholesterol (mg/dL)	165.0 ± 44.9	177.0 ± 42.6	0.548
HDLc (mg/dL)	45.0 ± 11.4	47.0 ± 15.4	0.745
LDLc (mg/dL)	91.6 ± 38.6	97.8 ± 39.4	0.726
Triglycerides (mg/dL)	143.1 ± 52.1	220.0 ± 158.7	0.174
Fast glucose (mg/dL)	115.9 ± 39.1	108.0 ± 15.1	0.584
Creatinine (mg/dL)	0.95 ± 0.14	0.96 ± 0.24	0.895
Uric acid (mg/dL)	5.6 ± 1.6	6.0 ± 2.0	0.594
TSH (mIU/L)	3.1 ± 1.1	2.3 ± 1.4	0.195
Ultrasensitive PCR (mg/dL)	0.42 ± 0.49	0.33 ± 0.37	0.626

AE, aerobic exercise session; IE, isometric exercise session; BMI, body mass index; MI, myocardial infarction; CAD, coronary artery disease; HDLc, high-density cholesterol; LDLc, low-density cholesterol; TSH, thyroid stimulating hormone; PCR, C-reactive protein. Student's t-test for independent samples or the chi-square test were used when applicable; *p* < 0.05. *Heavy consumption was defined as > 15 (males) and 10 (females) drinks/week (one drink contains 15 g of alcohol and is equivalent to 350 mL of beer, 150 mL of wine or 40 mL of spirits)

**Table 2:** Echocardiographic and carotid and vertebral artery measurements

	AE (n = 10)	IE (n = 10)	*p* value
Ejection fraction (Simpson method)	55.3 ± 8.7	55.9 ± 8.2	0.876
RPE			0.639
≥ 0.42 (cardiac remodeling)	3	4	
< 0.42 (normal)	7	6	
Left ventricular EDV (mL)	4.8 ± 0.5	4.8 ± 0.5	0.802
Left ventricular ESV (mL)	3.1 ± 0.5	3.1 ± 0.4	0.823
Left atrium (cm)	3.6 ± 0.4	3.8 ± 0.3	0.134
Diastolic dysfunction			0.572
None (normal function)	2	2	
Grade 1 (impaired relaxation)	7	7	
Grade 2 (pseudo-normal)	0	1	
Grade 3 (restrictive)	1	0	
Systolic pulmonary pressure			0.368
Normal (< 30 mmHg)	9	9	
Mild (30–50 mmHg)	1	0	
Moderate to severe (> 50 mmHg)	0	1	
Left ventricular mass (g)	83.4 ± 18.2	94.9 ± 22.1	0.219
Carotid artery status			0.506
1. < 50% stenosis	5	3	
2. 50–69% stenosis	4	5	
3. ≥ 70% stenosis	1	1	
4. Subocclusion	0	1	
Presence of atherosclerotic plaque			0.606
Yes	8	7	
No	2	3	

AE, aerobic exercise session; IE, isometric exercise session; RPE, relative parietal thickness; EDV, end-diastolic volume; ESV, end-systolic volume; carotid stenosis classification (PSV, peak systolic velocity; ICA, internal carotid artery): < 50% carotid stenosis: ICA PSV < 125 cm/s; carotid plaque with < 50% cross-sectional luminal narrowing; (2) 50%–69% carotid stenosis: ICA PSV 125–230 cm/s; plaque with ≥ 50% cross-sectional luminal narrowing; (3) ≥ 70% carotid stenosis: ICA PSV > 230 cm/s; plate with > 50% cross-sectional luminal narrowing; (4) carotid sub-occlusion: PSV variables; plate with large carotid sub-occlusion. Student's t-test for independent samples or the chi-square test were used when applicable; *p* < 0.05

**Table 3:** Medications use by intervention group

	AE (n = 10)	IE (n = 10)	*p* value
β-blockers	9	10	0.305
ASA	10	10	1.000
ACEIs/ARBs	10	10	1.000
Statins	10	10	1.000
Antiplatelet agents (clopidogrel)	9	10	0.305
Calcium channel blockers	2	1	0.589
Nitrates	6	4	0.422

AE, aerobic exercise session; IE, isometric exercise session; ASA, acetylsalicylic acid; ACEI, angiotensin converting enzyme inhibitors; ARBs, angiotensin II receptor blockers. Student's t-test for independent samples or the chi-square test were used when applicable; *p* < 0.05

**Table 4:** Clinical features of patients with acute myocardial infarction

	AE (n = 10)	IE (n = 10)	*p* value
Primary PCI			0.931
RCA (1 stent)	2	2	
ADA (1 stent)	3	3	
CXA (1 stent)	2	1	
RCA, ADA and CXA (3 stents)	2	2	
RCA, ADA (2 stents)	1	1	
ADA, ADA (2 stents)	0	1	
Primary PCI timing			0.146
6–12 h	3	4	
12–18 h	1	0	
18–24 h	0	2	
24–48 h	1	3	
> 48 h	5	1	

AE, aerobic exercise session; IE, isometric exercise session; PCI, percutaneous coronary intervention; RCA, right coronary artery; ADA, anterior descending artery; CXA, circumflex artery. Student's t-test for independent samples or the chi-square test were used when applicable; *p* < 0.05

The dataset supporting the conclusions of this article is included as Additional file 1.

## Results

A total of 32 patients receiving care at ICFUC outpatient clinic were eligible to participate in the study from February 2019 to July 2019 (recruitment and follow-up). Of these, 14 were excluded and 25 were recruited and invited to participate (Fig. 1). All study participants had history of ischemic heart disease and type I MI as described by Thygesen et al*.* (2018). There were data losses for five participants: one refused to undergo FMD and PWV assessments after the AE session; another one refused to participate in the IE session; and FMD results for the AE session (2 participants) and for the IE session (1 participant) were not included in the analysis due to imaging issues.

Table 1 shows the participants’ demographic characteristics and main cardiovascular risk factors. Briefly, they were mostly male (70%), 58.6 ± 9.0 years of age (AE group) and 56.0 ± 9.8 years (IE group), white and had complete high-school education. Fourteen volunteers (70%) were previously diagnosed with hypertension; 17 (85%) had family history of cardiovascular diseases; six (30%) had diabetes mellitus, six reported excessive alcohol use and 11 (55%) reported smoking.

Table 2 shows echocardiographic and carotid data of our sample. Relative wall thickness (RWT) was within the normal range for most participants (65%). Regarding diastolic dysfunction, 16 participants (80%) showed impaired relaxation of the myocardium and only two showed elevated pulmonary pressure. As for the carotid arteries, 12 (60%) showed carotid artery stenosis of 50% or more.

Table 3 summarizes medication use by intervention group. β-blockers were the most commonly used drugs as recommended. There was no difference between the two groups regarding optimized medication. Table 4 shows acute MI-related characteristics and they were similar in both groups.

**Fig. 2:**
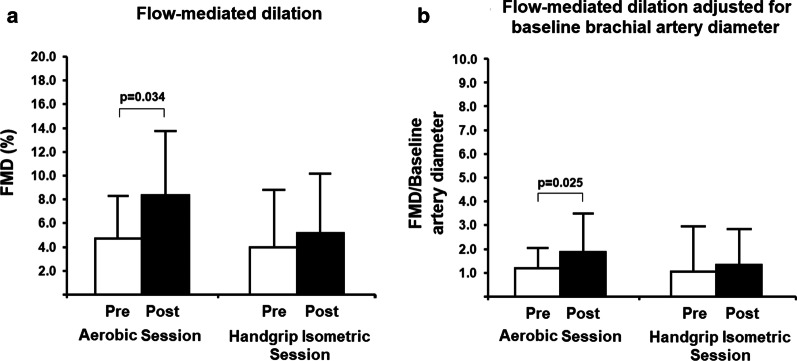
Pre- and post-exercise flow-mediated dilatation measurements. **a** shows flow-mediated dilation (FMD) before and after each exercise session. Since baseline brachial artery diameter (before cuff occlusion in FMD) was larger pre-exercise compared to post-exercise in the IE session, we adjusted FMD values for baseline brachial diameter. Thus, **b** shows FMD values adjusted for baseline brachial artery diameter. AE session (n = 10) and IE session (n = 10). Differences were tested by generalized estimating equations (GEE) with Bonferroni post-hoc test

Figure 2 shows FMD measurements. Interestingly, brachial artery diameter was reduced from baseline (before cuff occlusion) in the IE session after one exercise session (pre-exercise: 4.430 mm vs. post-exercise: 4.066 mm; *p* = 0.016) and there was no difference compared to the AE group (pre-exercise: 4.462 mm vs. post-exercise: 4.485 mm; *p* = 0.853). Peak diameter following hyperemia increased from baseline in the AE group (*p* = 0.028) and no change was observed in IE group (*p* = 0.091). Thus, FMD values increased from baseline in the AE group (Δ = 4.9%; *p* = 0.034), but there is no difference between groups in post-exercise moment (Fig. 2a). Yet, since baseline brachial artery diameter (before cuff occlusion) was smaller post-exercise than pre-exercise in the IE session, we adjusted FMD values for baseline brachial diameter following the literature [38]. After adjustment the effectiveness of AE remained (*p* = 0.025) with no change in the IE group (Fig. 2b).

**Fig. 3:**
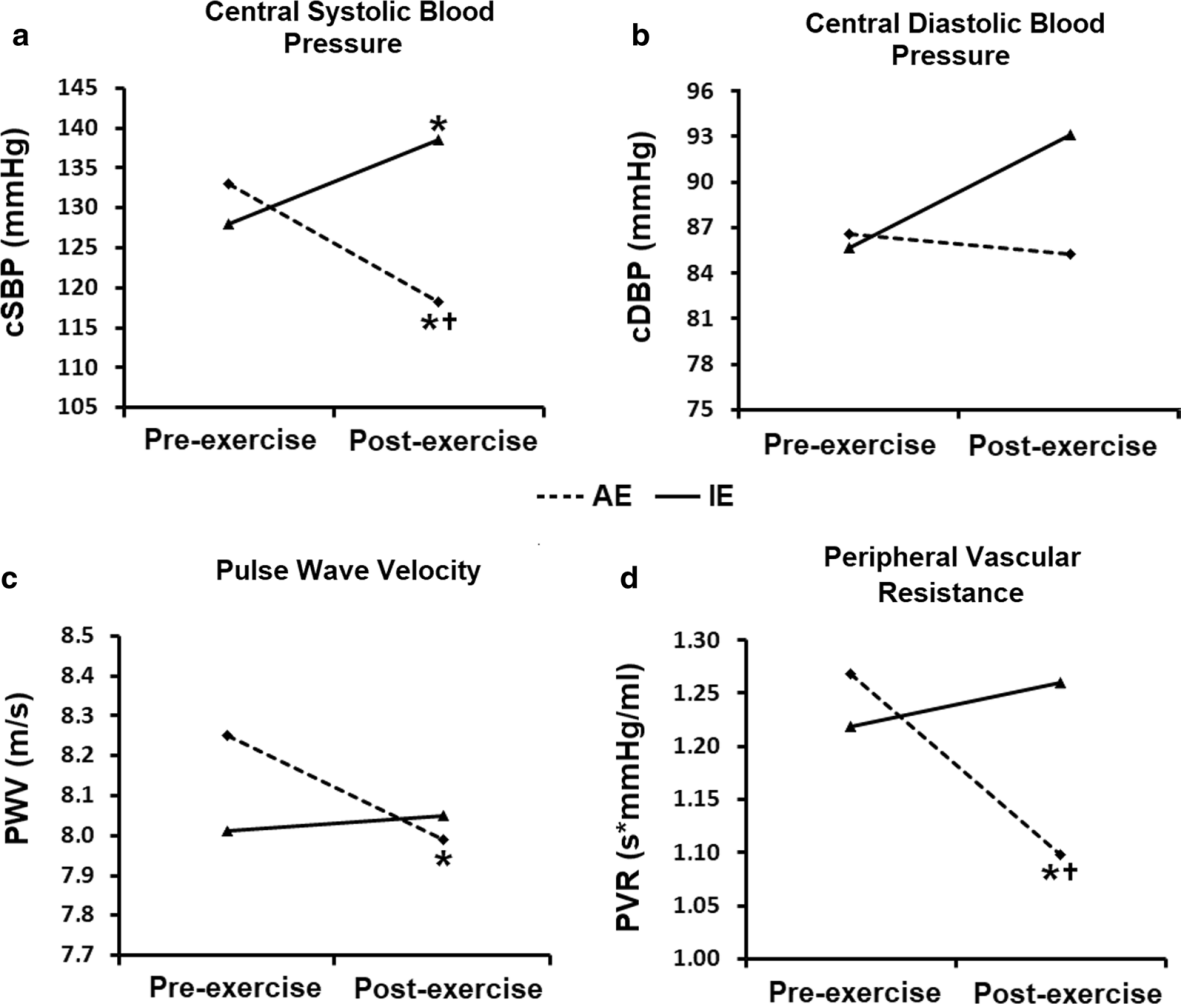
Pre- and post-exercise central blood pressure measurements. AE: aerobic exercise session (n = 10); IE: isometric exercise session (n = 10); cSBP: central systolic blood pressure; cDBP: central diastolic blood pressure; PWV: pulse wave velocity; PVR: peripheral vascular resistance. Differences in times, intervention sessions and session interactions were tested by generalized estimating equations (GEE) with Bonferroni post-hoc test. *p < 0.05 versus baseline within each intervention group; ^†^*p* < 0.05 versus IE at the same time point

Figure 3 presents central blood pressure measurements. AE effectively reduced cSBP post-exercise compared to IE (Δ = 20.1 mmHg, *p* = 0.011) as well as from baseline values (Δ = 14.9 mmHg, *p* = 0.002). After the exercise sessions, PWV values (Fig. 3c) were slightly reduced from baseline in the AE group (Δ = 0.61 m/s, *p* = 0.044), but not when compared to the IE group. PVR decreased in the AE group compared to the IE group (*p* = 0.050) and from baseline (*p* = 0.014) (Fig. 3d). Individual values are shown in the Additional file 2: Fig. 1S. Moreover, no difference in augmentation index corrected for heart rate of 75 bpm (AIX@75) was observed in both groups (AE: from 11.9 ± 4.1 to 15.5 ± 2.6%, *p* = 0.472; IE: from 10.8 ± 2.4 to 11.1 ± 3.0%, *p* = 0.903).

**Fig. 4:**
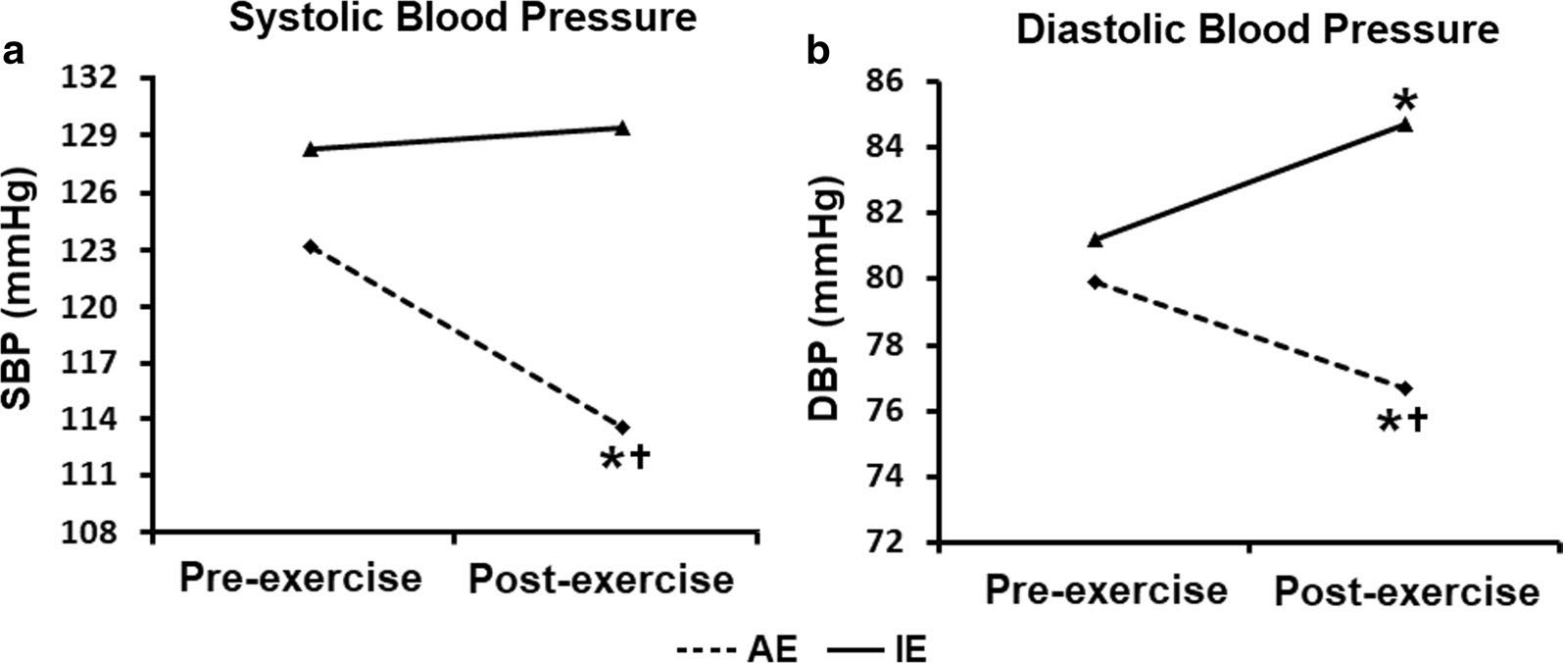
Pre- and post-exercise peripheral blood pressure measurements. AE: aerobic exercise session (n = 10); IE: isometric exercise session (n = 10); SBP: systolic blood pressure; DBP: diastolic blood pressure. Differences in times, intervention sessions and session interactions were tested by generalized estimating equations (GEE) with Bonferroni post-hoc test. *p < 0.05 versus baseline within each intervention session, ^†^*p* < 0.05 versus IE at the same time point

As for peripheral blood pressure (Fig. 4), systolic (SBP, *p* = 0.021) and diastolic (DBP, *p* < 0.001) were lower at the end of the session in the AE than the IE group. In addition, DBP increased from baseline after the IE session (*p* = 0.033). Individual peripheral blood pressure measures are shown in the Additional file 2: Fig. 2S.

Central blood pressure values were moderately correlated with PWV and PVR: cSBP and PWV (r = 0.610; *p* < 0.001) and cDBP and PVR (r = 0.418; *p* = 0.007). Peripheral blood pressure values were also moderately correlated with PWV and PVR: SBP and PWV (r = 0.593; *p* < 0.001), SBP and PVR (r = 0.628; *p* < 0.001), and DBP and PVR (r = 0.587; *p* < 0.001).

## Discussion

The main finding of our study was that, overall, one single session of AE improved endothelial function as assessed by FMD as well as central blood pressure measurements (cSBP/cDBP and PWV) in volunteers undergoing PCI after acute MI. Both peripheral blood pressure and vascular resistance decreased after the AE session. One session of IE did not prove effective in improving these same parameters; however, it was not associated with damaging effects on the cardiovascular system. It is important to stress that our findings do not challenge the recommendation of strength training for post-MI individuals since strength training consisting of dynamic and/or isometric exercises promotes health benefits, but rather that this population should perform it together with other exercise modalities, mainly aerobic exercises. The study findings are clinically relevant and support current physical exercise recommendations for post-MI individuals undergoing primary PCI.

Previous studies with individuals with impaired endothelial function due to aging and/or cardiovascular risk factors have demonstrated that a single AE session can improve endothelial function [39–41], which is consistent with our findings. Several mechanisms may explain improved endothelial function in response to AE, but the most widely studied mechanism is an increase in nitric oxide (NO) bioavailability that promotes an improvement in vasodilatory capacity [42]. Increased blood flow in response to one session of AE demands induces shear stress leading to higher NO bioavailability. Interestingly, different exercise modalities are associated with different patterns of luminal shear stress [5] resulting in distinctive vascular function responses [43]. During IE there is sustained mechanical compression of active muscles and muscular relaxation at the end of the movement determines an increase in blood flow leading to shear stress. Thus, while AE stimulus is continuous, IE stimulus is intermittent (muscular mechanical compression followed by blood flow release). Interestingly, it has been postulated that one session of IE increases sympathetic activity [44] so that arterial vasoconstriction might be greater than NO vasodilatory capacity. One finding of our study supporting this hypothesis is that after a single exercise session, resting brachial artery diameter was smaller (vasoconstricted) than pre-exercise diameter and it could explain our FMD results in the IE group. It is crucial to understand whether this is a transient effect, i.e., an acute/subacute effect in response to exercise, and if it has any long-term adverse vascular effects, particularly in patients undergoing primary PCI after myocardial infarction.

As for central blood pressure measurements, one session of AE reduced PWV while one session of IE did not have any central effects. Evidences has shown that shorter exercise duration (as our protocol, 30 min) was associated with favorable vascular effects, and longer exercise (~ 60 min) had adverse effects on vascular stiffness, mainly in older coronary patients [45]. PWV as a measurement of aortic stiffness is an independent predictor of adverse cardiac and cerebrovascular outcomes in post-MI patients undergoing primary PCI including death, nonfatal reinfarction, congestive heart failure and stroke. Based on our findings, we stress the importance of AE—even a single exercise session—for post-MI rehabilitation. They also suggest that IE does not seem an adequate approach for reducing central blood pressure as it did not prove effective in decreasing PWV and central blood pressure measurements, though it did not have negative effects. Evidence from prior studies shows that long-term high-intensity strength training can increase PWV in individuals with increased arterial stiffness [17]. These authors as well as others have reported that mild to moderate strength exercise does not reduce arterial stiffness [14–16]. However, they have assessed dynamic exercises only, not isometric.

Considering there is a decrease in central systolic pressure and PWV as well as an increase in endothelium-dependent vasodilatory capacity, it would be also expected a decrease in PVR following AE, and this evidence may be associated with lower peripheral blood pressure in our study. SBP decreased by around 6 mmHg within 40 min of AE. Our results are clinically relevant and consistent with studies indicating a hypotensive effect after AE with SBP reduction by 5 to 7 mmHg [46]. In addition, IE increased cSBP and decreased baseline brachial artery diameter, but it did not have any effects on PWV, FMD, peripheral SBP and PVR. In particular with regard to peripheral SBP, other studies have not shown decreased BP values [20, 47] in response to a single session of isometric handgrip exercise. It should be noted that our study evaluated one exercise session only and that continuing isometric handgrip training could lead to a reduction in SBP (− 5.4 mmHg) and DBP (− 2.4 mmHg) as shown in a recent meta-analysis [48].

This present study has some limitations. Our sample was small (n = 20) because we sought to include volunteers with very similar clinical characteristics (post-MI adults undergoing PCI with the placement of drug-eluting stents). After achieving the predicted sample size, there were five losses on the intervention day (one with missing imaging study results and four dropouts due to long waiting times). We achieved 84% of the predicted sample size with 70% statistical power for the primary outcome. Thus, our small sample precludes sub-analyses by gender, carotid artery status, ejection fraction among others. Another limitation is that we did not include a control group (no exercise); however, our purpose was to compare one IE session versus one AE session [primary strategy in cardiac rehabilitation guidelines and recommendations [9–12]]. Finally, it should be stressed that various exercise modalities involve different muscle masses. AE uses large muscle groups (area and mass) while IE uses smaller muscle groups. These differences could be further investigated using lower-limb IE (e.g., exercise in a stretching extensor bench).

## Conclusions

When compared to single bout of AE, one session of IE apparently does not have vascular and hemodynamic benefits in patients undergoing primary PCI after MI. Nevertheless, our intervention of IE did not apparently show any negative vascular or hemodynamic effects, as reported in other populations [23–25]. Given the small sample size, our findings do not challenge the importance of IE strength training, but they draw attention to the fact that this exercise modality should not be used as a single approach for vascular improvement in post-MI individuals with a profile similar to our sample.

## Supplementary Information

**Additional file 1.** The dataset supporting the results and conclusions of this article.

**Additional file 2.** Individual values regarding behavior pattern of central and peripheral blood pressure measurements.

## Data Availability

The dataset supporting the conclusions of this article is included within the article and its Additional file 1.

## Abbreviations

ACE: Angiotensin-converting enzyme
ACEI: Angiotensin converting enzyme inhibitors
ADA: Anterior descending artery
AE: Aerobic exercise
ARBs: Angiotensin II receptor blockers
ASA: Acetylsalicylic acid
BMI: Body mass index
CAD: Coronary artery disease
CCA: Common carotid artery
cDBP: Central diastolic blood pressure
cSBP: Central systolic blood pressure
CXA: Circumflex artery
DBP: Diastolic blood pressure
EDV: Left ventricular end-diastolic volume
EDVe: End-diastolic velocity
ESV: Left ventricular end-systolic volume
ESVI and EDVI: ESV and EDV adjusted for body surface area
FMD: Flow-mediated dilation
HDLc: High-density cholesterol
ICA: Internal carotid artery
IE: Isometric exercise
IPAq: International Physical Activity Questionnaire
HR: Heart rate
LDLc: Low-density cholesterol
LV: Left ventricular
MI: Myocardial infarction
MVC: Maximal voluntary contraction
NO: Nitric oxide
PCI: Percutaneous coronary intervention
PCR: C-reactive protein
PSV: Peak systolic velocity
PVR: Peripheral vascular resistance
PWV: Pulse wave velocity
RCA: Right coronary artery
RPE: Relative parietal thickness
RWT: Relative wall thickness
SBP: Systolic blood pressure
TIA: Transient ischemic attack
TSH: Thyroid stimulating hormone
