# Supplementary material for: Aerobic exercise, but not isometric handgrip exercise, improves endothelial function and arterial stiffness in patients with myocardial infarction undergoing coronary intervention: a randomized pilot study
Source: BMC Cardiovasc Disord. 2021 Feb 17;21:101. doi: 10.1186/s12872-021-01849-2 (PMC7887833; doi:10.1186/s12872-021-01849-2)
Supplement: Supplementary file 2 — Additional file 2. Individual values regarding behavior pattern of central and peripheral blood pressure measurements. [file 12872_2021_1849_MOESM2_ESM.pdf]

Supplementary material

Aerobic exercise, but not isometric handgrip exercise, improves endothelial function and arterial stiffness in patients with myocardial infarction undergoing coronary intervention: a randomized pilot study

Running title: Exercise and vascular function

Daniel P Kollet[1], Ana Beatriz Marenco[1], Nathan L Bellé[1], Eduardo Barbosa[1], Liliana Boll[1], Bruna Eibel[1], Gustavo Waclawovsky[1], Alexandre M Lehnen[1*]

1. Institute of Cardiology of Rio Grande do Sul/University Foundation of Cardiology, Porto Alegre, Rio Grande do Sul, Brazil.

* https://orcid.org/0000-0002-5912-8020

[1] * **Corresponding Author**
Dr Alexandre Machado Lehnen
Instituto de Cardiologia do Rio Grande do Sul/Fundação Universitária de Cardiologia
Unidade de Pesquisa, 3°Andar
Av. Princesa Isabel, 395 Santana, 90620-001 Porto Alegre – RS Brazil
amlehnen@gmail.com

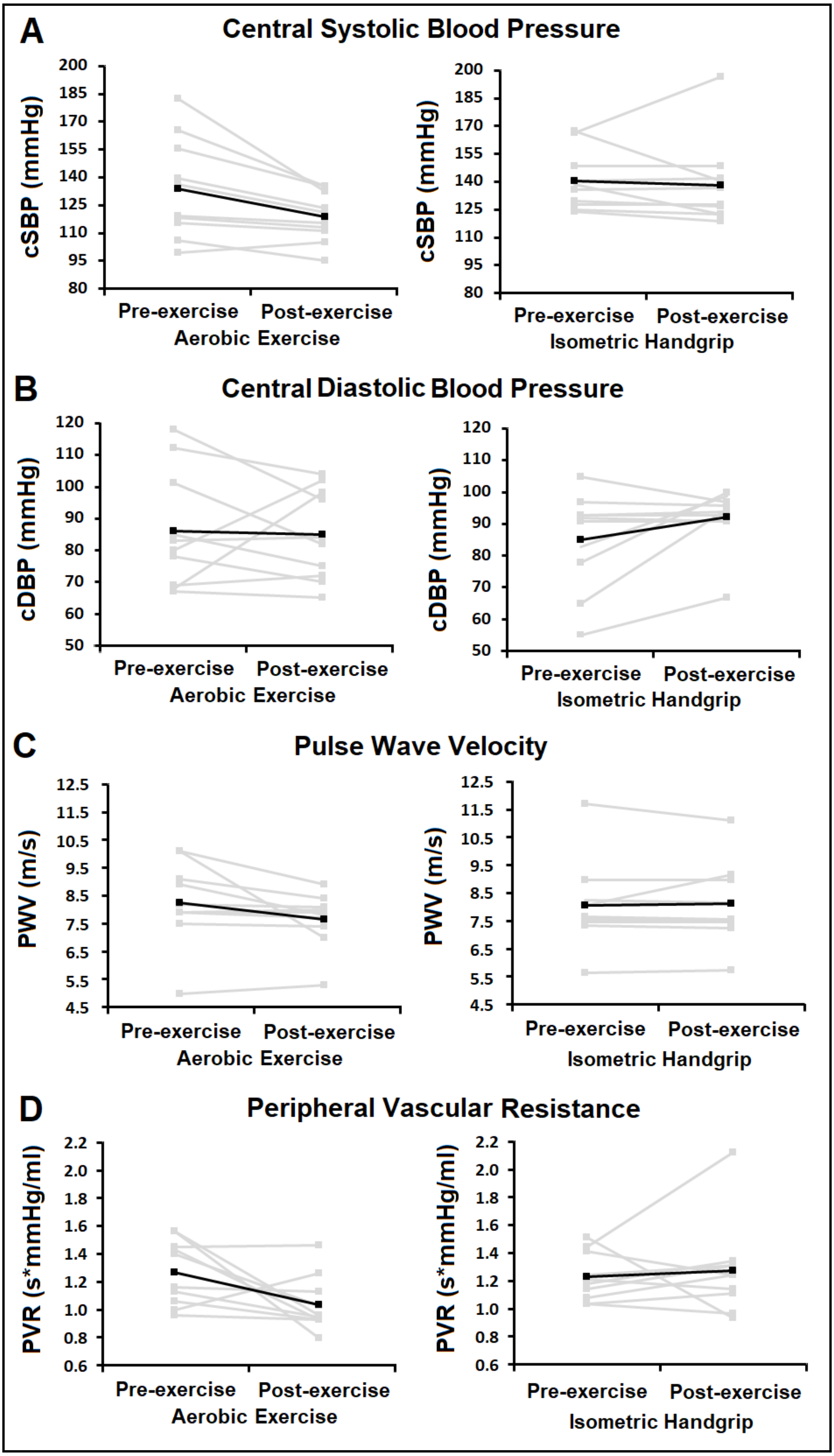
A
Central Systolic Blood Pressure
cSBP (mmHg)
Pre-exercise
Post-exercise
Aerobic Exercise
Isometric Handgrip
B
Central Diastolic Blood Pressure
cDBP (mmHg)
C
Pulse Wave Velocity
PWV (m/s)
D
Peripheral Vascular Resistance
PVR (s*mmHg/ml)

Figure 1S – Behavior pattern of central blood pressure measurements. cSBP: central systolic blood pressure; cDBP: central diastolic blood pressure; PWV: pulse wave velocity; PVR: peripheral vascular resistance. The gray lines represent individual values and the black line is the mean of individual values

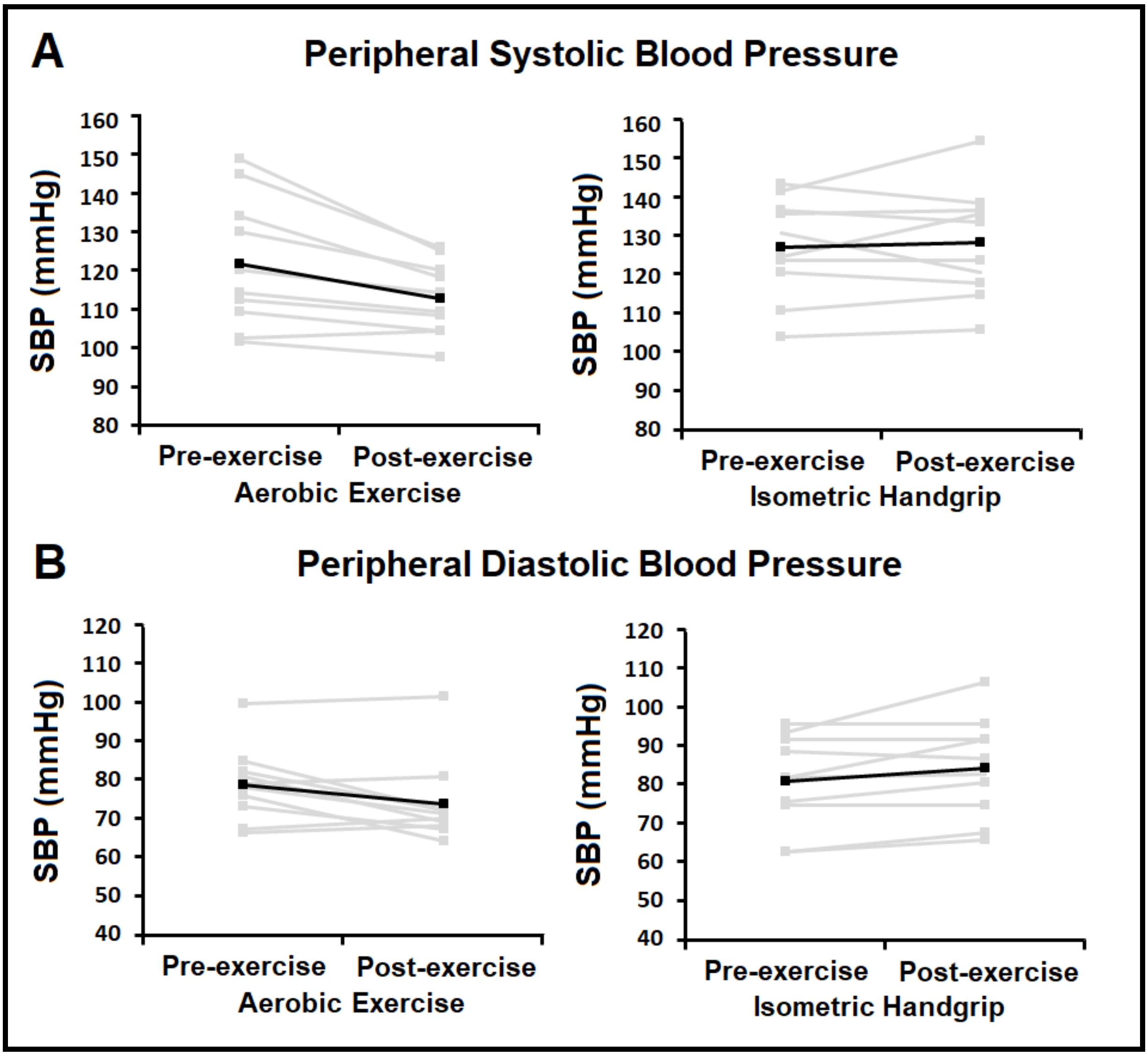

Figure 2S – Behavior pattern of peripheral blood pressure measurements. SBP: systolic blood pressure; DBP: diastolic blood pressure. The gray lines represent individual values and the black line is the mean of individual values.
